# Racial and Ethnic Differences in Cannabis Use Following Legalization in US States With Medical Cannabis Laws

**DOI:** 10.1001/jamanetworkopen.2021.27002

**Published:** 2021-09-27

**Authors:** Silvia S. Martins, Luis E. Segura, Natalie S. Levy, Pia M. Mauro, Christine M. Mauro, Morgan M. Philbin, Deborah S. Hasin

**Affiliations:** 1 Department of Epidemiology, Columbia University Mailman School of Public Health, New York, New York; 2 Department of Biostatistics, Columbia University Mailman School of Public Health, New York, New York; 3 Department of Sociomedical Sciences, Columbia University Mailman School of Public Health, New York, New York; 4 Department of Psychiatry, Columbia University Irving Medical Center, New York, New York; 5 New York State Psychiatric Institute, New York, New York

## Abstract

**Importance:**

Little is known about changes in cannabis use outcomes by race and ethnicity following the enactment of recreational cannabis laws (RCLs).

**Objectives:**

To examine the association between enactment of state RCLs and changes in cannabis outcomes by race and ethnicity overall and by age groups in the US.

**Design, Setting, and Participants:**

This cross-sectional study used restricted use file data from the National Surveys of Drug Use and Health between 2008 and 2017, which were analyzed between September 2019 and March 2020. National survey data included the entire US population older than 12 years.

**Main Outcomes and Measures:**

Self-reported past-year and past-month cannabis use and, among people that used cannabis, daily past-month cannabis use and past-year *Diagnostic and Statistical Manual of Mental Disorders* (Fifth Edition) cannabis use disorder. Multi-level logistic regressions were fit to estimates changes in cannabis use outcomes by race and ethnicity overall and by age between respondents in states with and without enacted RCLs, controlling for trends in states with medical cannabis laws or no cannabis laws.

**Results:**

A total of 838 600 participants were included for analysis (mean age, 43 years [range, 12-105 years]; 434 900 women [weighted percentage, 51.5%]; 511 900 participants (weighted percentage, 64.6%) identified as non-Hispanic White, 99 000 (11.9%) as non-Hispanic Black, 78 400 (15.8%) as Hispanic, and 149 200 (7.6%) as other (including either Native American, Pacific Islander, Asian, or more than 1 race or ethnicity). Compared with the period before RCL enactment, the odds of past-year cannabis use after RCL enactment increased among Hispanic (adjusted odds ratio [aOR], 1.33; 95% CI, 1.15-1.52), other (aOR, 1.31; 95% CI, 1.12-1.52), and non-Hispanic White (aOR, 1.21; 95% CI, 1.12-1.31) populations, particularly among those aged 21 years or more. Similarly, the odds of past-month cannabis use increased among Hispanic (aOR, 1.43; 95% CI, 1.22-1.69), other (aOR, 1.43; 95% CI, 1.20-1.70), and non-Hispanic White (aOR, 1.24; 95% CI, 1.13-1.35) populations after RCL enactment. No increases were found in the odds of past-year or past-month cannabis use post-RCL enactment among non-Hispanic Black individuals or among individuals aged 12 to 20 years for all race and ethnicity groups. In addition, among people who used cannabis, while no increases were found in past-month daily cannabis in any racial or ethnic group, the odds of cannabis use disorder increased post-RCL among individuals categorized as other overall (aOR, 1.45; 95% CI, 1.07-1.95), but no increases were found by age group.

**Conclusions and Relevance:**

Changes in cannabis use by race and ethnicity that may be attributable to policy enactment and variations in recreational policy provisions should be monitored. To ensure that the enactment of recreational cannabis laws truly contributes to greater equity in outcomes and adheres to antiracist policies, monitoring unintended and intended consequences that may be attributable to recreational cannabis use and similar policies by race and ethnicity is needed.

## Key Points

**Question:**

Is the legalization of recreational cannabis in the US associated with changes in cannabis use outcomes and cannabis use disorder across racial and ethnic groups?

**Findings:**

In this cross-sectional study analyzing repeated yearly surveys of US adults conducted from 2008 to 2017, living in a state after enactment of recreational cannabis laws was associated with increases in the odds of cannabis use within the past year and past month among Hispanic and non-Hispanic White individuals (as well as individuals identifying as Native American, Pacific Islander, Asian, or more than 1 race) compared with the period before the passage of recreational use laws; there were no increases among non-Hispanic Black individuals.

**Meaning:**

Cannabis legalization is generally associated with increased use of cannabis and not associated with frequent use or use disorder among cannabis users, including among members of demographic subgroups most affected by criminalization.

## Teaser

This cross-sectional study compares self-reported rates of cannabis use before and after the enactment of recreational use laws in US states by race and ethnicity.

## Introduction

Cannabis laws are changing rapidly in the US. By January 2021, 15 states and Washington, DC, had fully legalized cannabis use for adults aged 21 years or more and an additional 21 states had legalized medical cannabis.^1^ Research indicates that enactment of medical cannabis laws (MCLs) is associated with increases in the odds of past-year and daily cannabis use among adults aged 18 years or more,^2,3,4^ but no changes have been observed for adolescents between ages 12 and 17 years.^5,6^ Research has also identified modest increases in the adjusted prevalence of past-month cannabis use (2008, 5.65% vs 2016, 7.10%) and past-year cannabis use disorder (CUD) (2008, 0.90% vs 2016, 1.23%) among adults aged 26 years or more after enactment of legal adult use (ie, recreational cannabis laws [RCLs]) but not among adults aged 26 years or more that used cannabis.^7^

RCL enactment is often framed as an issue of social and racial justice. Historically, regulation and criminalization of substances in the US has targeted substances associated with underserved racial and ethnic minority populations. The legacy of racism and discrimination imbedded in cannabis legislation is reflected in the stark racial disparities in cannabis-related arrests and incarcerations. For example, in 2018 the lifetime prevalence of cannabis use was lower for Black (45.3%) than White (53.6%) adults aged 18 years or older,^8^ but Black individuals were 3.64 times more likely to be arrested for cannabis possession.^9^ In states with RCLs, overall rates of cannabis arrests have decreased but Black and Hispanic individuals are still more likely to be arrested than their White counterparts.^9,10^ Communities of color (Black and Hispanic populations, as well as some Asian subgroups) may be more likely than White populations to experience negative consequences of legalization, including increased frequent cannabis use and CUD.^11^ Such unintended consequences could occur because of structural factors informed by the legacies of US racism, for example locating dispensaries (which have been tied to increased CUD^12,13^) in neighborhoods with a majority racial or ethnic minority population.

If RCLs are achieving some measure of greater racial equity, the potential negative consequences of increased cannabis availability should not disproportionately affect Black or Hispanic populations. To our knowledge, no study has examined whether cannabis legalization differentially affects cannabis use outcomes by race and ethnicity. Investigating potential differences by race and ethnicity of cannabis use, daily use, and CUD post-RCL enactment is important for evaluating these policies as racial health equity measures^14^ and for identifying groups in need of intervention, whether through the more equitable provision of substance use prevention and treatment resources or through closer scrutiny of discriminatory law enforcement. To this end, we examined changes in cannabis outcomes before and after RCL enactment by race, ethnicity, and age. We hypothesized that past-year and past-month cannabis use would increase equally for all race and ethnicity categories (particularly for adults aged 21 years or more) and that no changes would be observed for daily cannabis use and CUD among individuals using cannabis by race or ethnicity.

## Methods

### Study Sample

The National Survey on Drug Use and Health (NSDUH) is an annual household survey of the US noninstitutionalized population aged 12 and older (approximately 70 000 individuals annually). The survey uses multistage probability sampling to produce representative national and state estimates of substance use–related behaviors and mental health conditions. Interviews are conducted by trained staff using computer-assisted personal interviewing and audio computer-assisted self-interviewing to increase privacy and accurate reporting of sensitive information. The NSDUH includes survey weights to generate representative population estimates and account for the probability of selection, nonresponse, coverage, and extreme weights. Interview response rates between 2008 and 2017 varied between 67% and 76%.^15^

We used restricted-use NSDUH data between 2008 and 2017; 2017 was the most recent year with analyses available for restricted data. The study included 838 600 respondents. Reported sample sizes were rounded to the nearest hundred and values less than 100 were suppressed in accordance with Substance Abuse and Mental Health Services Administration requirements. This study was approved by the Columbia University institutional review board. Patients provided written informed consent as part of NSDUH, and this study was considered exempt from additional informed consent because data were deidentified. This manuscript was prepared according to the Strengthening the Reporting of Observational Studies in Epidemiology (STROBE) reporting guideline for cross-sectional studies.

### Measures

Our primary exposure was residence in a state with RCL (ie, legal adult cannabis use) enactment. Information on the enactment dates of RCLs and MCLs were obtained from the Marijuana Policy Project^16^ and ProCon.org^1^ and were based on the specific language of the statute, accounting for any necessary conditions for the law to go into effect. The dates used in our analysis are summarized in eTable 1 in the Supplement.

State cannabis law status were defined using a fixed categorical variable for descriptive analyses and a time-varying variable for regression models. For descriptive purposes, we categorized status as: (1) never MCL/RCL for states that never had enacted MCL or RCL during the study period, (2) MCL only/no RCL for states that had enacted MCL, and (3) ever RCL for states with an enacted RCL (Table 1). The time-varying indicator of state MCL and RCL status compared the date on which a participant was interviewed with the enacted MCL and/or RCL date in their state of residence. For example, if the interview date was later than the RCL effective date then participants were classified as living in a state where RCLs were enacted, and they were classified as otherwise if interview date was earlier than RCL date of enactment. Participants were classified into 1 of 6 possible categories: never MCL/RCL, before MCL/never RCL, after MCL/never RCL, before MCL/before RCL, after MCL/before RCL, and after MCL/after RCL (Figure). Example code for creating this time-varying indicator of cannabis legalization status is provided in eAppendix in the Supplement.

**Table 1.** Distribution of Demographic Characteristics, Cannabis Outcomes, and State-Level Covariates Between States That Differ by Cannabis Law Status

	Participants, No. (weighted %)		
	No cannabis laws (n = 191 600)	Medical cannabis laws only (n = 490 400)	Recreational cannabis laws (n = 156 600)
**Individual-level factors**			
Sex^a^			
Women	99 900 (51.7)	254 300 (51.5)	80 700 (51.0)
Men	91 700 (48.4)	236 100 (48.6)	76 000 (49.0)
Age, mean (range), y	43 (12-102)	43.77 (12-105)	43.06 (12-100)
12-20	78 100 (15.0)	201 900 (14.4)	64 300 (14.8)
21-30	48 600 (16.5)	123 400 (16.1)	39 800 (16.6)
31-40	22 400 (15.6)	55 800 (15.2)	18 100 (15.8)
41-50	19 600 (16.2)	50 900 (16.1)	16 200 (16.2)
51-64	13 400 (21.1)	34 100 (21.5)	10 900 (21.1)
≥65	9500 (15.6)	24 300 (16.8)	7400 (15.5)
Race or ethnicity			
Non-Hispanic White	122 100 (66.3)	299 200 (64.6)	90 600 (58.7)
Non-Hispanic Black	28 900 (15.6)	55 500 (10.2)	14 600 (6.5)
Hispanic	12 000 (4.6)	48 900 (8.7)	17 500 (12.2)
Other^b^	28 600 (13.5)	86 700 (16.4)	33 900 (22.6)
Nativity			
US-born	175 400 (89.5)	429 500 (82.6)	135 200 (78.2)
Born outside the US	16 100 (10.5)	60 900 (17.4)	21 500 (21.8)
Education			
Less than high school	21 100 (16.0)	48 100 (13.3)	15 400 (13.6)
High school graduate	41 000 (29.3)	103 400 (27.9)	31 800 (25.4)
Some college	42 600 (27.8)	105 700 (27.7)	33 800 (28.5)
College graduate	30 000 (26.9)	87 000 (31.1)	29 000 (32.5)
Annual income, $			
<20 000	44 100 (19.3)	103 800 (17.3)	33 500 (16.8)
20 000-49 999	64 400 (33.4)	154 300 (30.8)	49 400 (30.2)
50 000-74 999	31 400 (17.4)	78 800 (16.6)	24 600 (16.2)
≥75 000	51 600 (29.9)	153 500 (35.4)	49 200 (36.8)
Urbanicity			
Large metropolitan	60 600 (42.5)	247 600 (61.0)	80 600 (64.8)
Small metropolitan	77 100 (35.5)	163 300 (27.9)	52 400 (27.1)
Nonmetropolitan	53 900 (21.9)	79 400 (11.1)	23 600 (8.2)
Insurance			
Private	116 900 (65.2)	302 800 (67.2)	95 000 (66.3)
Medicaid/SCHIP	33 500 (8.6)	104 400 (12.3)	35 800 (13.4)
Medicare	12 500 (20.3)	31 600 (20.6)	9600 (18.8)
Military	8400 (6.1)	15 900 (4.4)	5100 (4.1)
Other	4200 (1.8)	12 700 (2.1)	4100 (2.2)
None	31 700 (16.5)	63 100 (12.4)	19 800 (12.9)
Any health insurance	159 900 (83.5)	427 300 (87.6)	136 900 (87.1)
Substance use			
Past year cannabis use	28 900 (10.3)	94 000 (13.5)	34 900 (16.1)
Past month cannabis use	16 500 (6.1)	57 400 (8.5)	22 100 (10.6)
Past month daily cannabis use^c^	6400 (2.5)	22 500 (3.6)	9000 (4.6)
*DSM-5* cannabis use disorder^d^	4800 (1.3)	15 100 (1.6)	5400 (1.8)
**State-level factors**			
Demographics, mean (range), %			
Men	49.2 (48.3-51.0)	49.1 (47.1-52.0)	49.6 (47.1-52.0)
White	73.8 (59.1-93.9)	72.5 (24.3-96.9)	68.9 (30.8-96.9)
10-24 y	21.3 (20.1-23.9)	20.7 (18.6-28.4)	21.2 (18.6-23.4)
>25 y with at least high school	16.7 (7.7-27.1)	14.9 (8.2-25.2)	15.9 (8.9-23.2)
Unemployment rate	5.9 (2.5-8.0)	6.5 (2.7-9.4)	7.2 (2.9-9.4)
Median household income, mean (range), $	45 313 (31 330-60 674)	50 858 (29 696-68 854)	53 424 (37 240-64 576)

Abbreviations: *DSM-5*, *Diagnostic and Statistical Manual of Mental Disorders* (Fifth Edition); SCHIP, State Children’s Health Insurance Program.

^a^ Sex reported in terms of gender in the National Survey on Drug Use and Health study.

^b^ Because small numbers of participants endorsed several of the listed categories, we recategorized Native American, Pacific Islander, Asian American, and more than 1 race as other race or ethnicity.

^c^ Daily cannabis use is the use of cannabis almost daily (20 or more days) in the past month among cannabis users.

^d^ Past-year cannabis use disorder was calculated among people who used cannabis.

**Figure.**
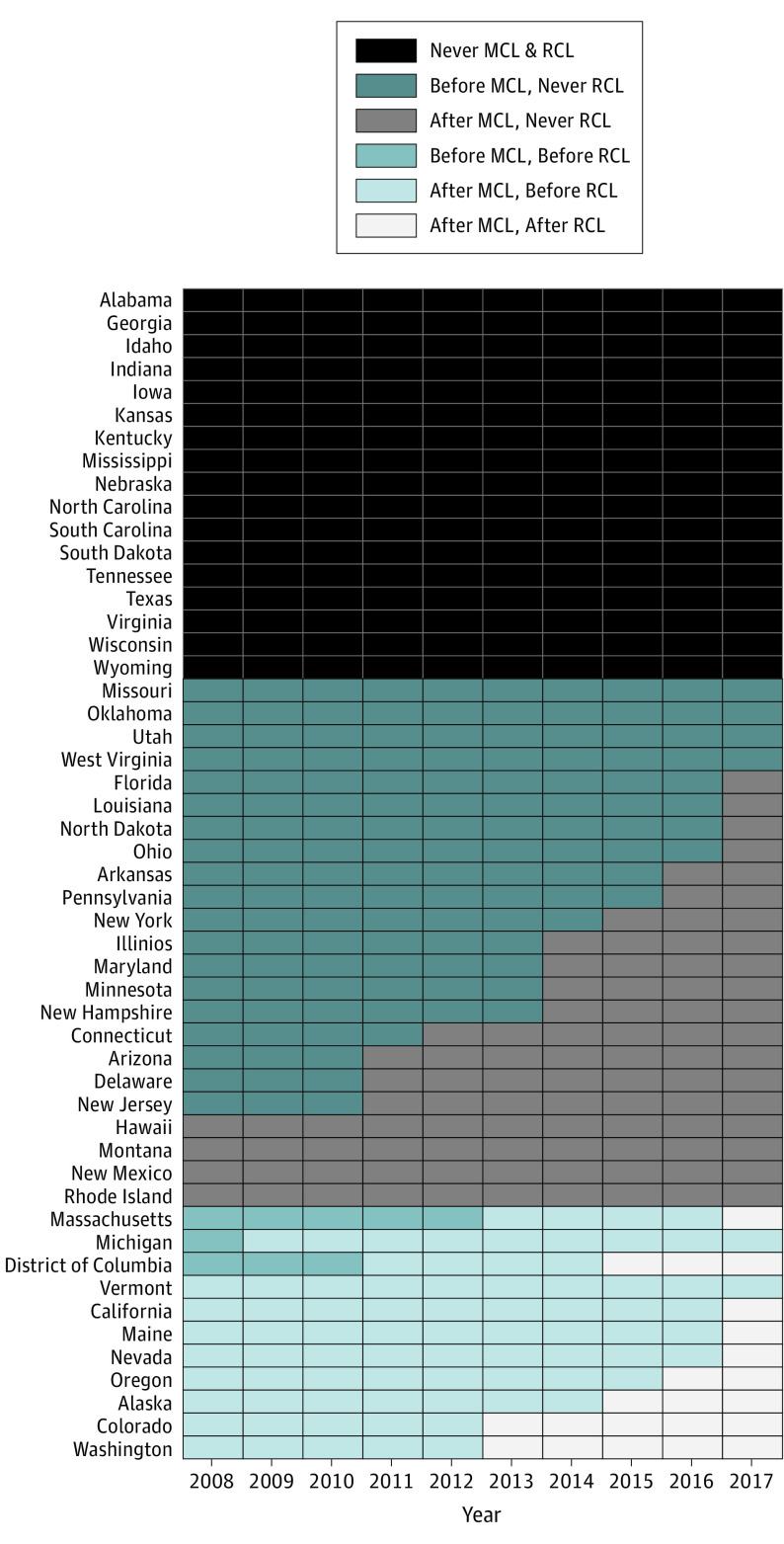
Enactment of Medical and Recreational Cannabis Laws in Each US State From 2008 to 2017 For descriptive purposes, status was categorized as: (1) never medical cannabis laws (MCLs) or recreational cannabis laws (RCLs) for states that never had enacted MCL or RCL during the study period, (2) MCL only/no RCL for states that had enacted MCL, and (3) ever RCL for states with an enacted RCL. The time-varying indicator of state MCL and RCL status compared the date on which a participant was interviewed with the enacted MCL and/or RCL date in their state of residence. For example, if the interview date was later than the RCL effective date then participants were classified as living in a state where RCLs were enacted, and they were classified as otherwise if interview date was earlier than RCL date of enactment.

All 9 states with enacted RCLs during the study period had previously enacted MCLs. As described below, for regression analyses we focused on the period after an MCL was enacted to isolate the effects of RCLs beyond effects of existing MCLs, comparing cannabis outcomes in the period before RCLs (after MCL/before RCL) were enacted with the period after RCLs were enacted (after MCL/after RCL).

Cannabis outcomes included past-year and past-month cannabis use in the general population, past-month daily cannabis use among people with past-month cannabis use, and past-year CUD among people with past-year cannabis use. Daily use was defined as using cannabis for 20 or more days in the past month, as defined elsewhere.^3^ To create the *Diagnostic and Statistical Manual of Mental Disorders* (Fifth Edition) (*DSM-5*) proxy variable, we followed the work of Compton et al^17^ by using by using the *DSM-IV* individual items asked in the NSDUH to approximate the 10 criteria used for the *DSM-5* diagnosis (removing the legal problems criterion and excluding cravings and withdrawal, which were not collected in the NSDUH) (eTable 2 in the Supplement). Individuals endorsing 2 or more of the 10 CUD criteria were coded as having a *DSM-5*-proxy CUD diagnosis.^17^

Indicators of race and ethnicity and age groups (ages 12-20, 21-30, 31-40, and ≥41 years) were used as effect modifiers of enacted RCLs on cannabis outcomes. Racial and ethnic groups were based on NSDUH’s race and ethnicity variable (non-Hispanic White, non-Hispanic Black, Hispanic, Native American, Pacific Islander, Asian, and more than 1 race), constructed from participants’ choice of the race group that best described them, and 2 separate indicators of more than 1 race and Hispanic ethnicity. Because small numbers of participants endorsed several of the listed categories, we recategorized Native American, Pacific Islander, Asian, and more than 1 race as other race or ethnicity.

We included factors at individual and state levels as potential confounding covariates. Individual-level covariates included gender, survey year, nativity, total family income (stratified as less than $20 000, $20 000 to $49 999, $50 000 to $74 999, and more than $75 000), and urbanicity. State-level factors were drawn from the 2010 US Census and included the proportion of each state’s population that was White, male, aged 10 to 24 years, aged 25 years or older with at least a high school education, state unemployment rates, and median household income.

### Statistical Analysis

We performed analyses between September 2019 and March 2020. We first calculated the weighted annual prevalence of each cannabis-related outcome by cannabis law enactment status (ie, never MCL/RCL, MCL only/no RCL, and ever RCL) by combining weighted counts and population totals for states belonging to each category of enactment status in each survey year. Prevalence variances were estimated using Taylor linearization, which incorporates variables related to the NSDUH complex survey design to account for clustering.^18^ Prevalence was estimated by race and ethnicity overall and by age group.

We then tested the association of enacted RCLs with cannabis outcomes by comparing the odds of each cannabis outcome during the period after MCL/before RCL with the period after MCL/after RCL by race and ethnicity overall and stratified by age groups. We used separate multilevel logistic regressions with state-level random intercepts, individual and state covariates, and 2-way and 3-way interactions between the time-varying RCL exposure, race and ethnicity, and age group. We used random state-level intercepts in our models to account for the NSDUH design, in which states were used as strata and clusters were sampled within states. We used data from all 50 states, including states without MCL and/or enacted RCL, to control for time trends in cannabis outcomes between 2008 and 2017. Survey weights were not included in our models because we included all individual-level indicators related to sampling design as covariates.^19^ For our main contrast of interest, we computed adjusted odds ratios (aOR) and 95% CIs comparing the categories after RCL enactment (after RCL/after MCL) vs before RCL enactment (after MCL/before RCL). This allowed us to estimate the change from before to after RCL enactment above and beyond any effects of MCL enactment using a method similar to a difference-in-difference approach.^7^ Similar to other research examining changes over time in cannabis outcomes, we included year as a continuous variable using a piecewise spline function of year with a knot at 2011.^7^

We conducted sensitivity analyses with e-values to evaluate the potential impact of time-varying unmeasured confounding^20,21,22^ on our results. Small e-values (values closer to 1.0) suggest that unmeasured confounding may account for observed associations; larger e-values indicate results that are increasingly robust to unmeasured confounding. E-values were obtained using the EValue package in R software.^21^

Per NSDUH requirements, all analyses were conducted at the Census Bureau’s New York Regional Data Center using the Linux-based R statistical software package 3.5.2 (R Project for Statistical Computing). All output was reviewed by Substance Abuse and Mental Health Services Administration staff to ensure that confidentiality guidelines were followed. R packages used for data analysis and eFigures were survey,^23^ lme4,^24^ and ggplot2.^25^ Statistical significance was determined by 95% CIs.

## Results

Of 838 600 total participants, 511 900 (weighted percentage, 64.6%) were non-Hispanic White, 99 000 (11.9%) non-Hispanic Black, 78 400 (15.8%) Hispanic, and 149 200 (7.6%) other race or ethnicity; 434 900 (51.5%) were women and the sample mean age was 43 years (range, 12-105 years) (Table 1).

### Racial and Ethnic Differences in RCL Enactment

Prevalence of past-year cannabis use increased after RCL/after MCL among Hispanic (11.7% to 15.0%; aOR, 1.33; 95% CI, 1.15-1.52), other (14.8% to 18.5%; aOR, 1.31; 95% CI, 1.12-152), and White (16.6% to 19.4%; aOR, 1.21; 95% CI, 1.12-1.31) participants (Table 2). Past-month cannabis use increased after RCL/after MCL among Hispanic (6.4% to 8.9%; aOR, 1.43; 95% CI, 1.22-1.69), other (8.8% to 12.1%; aOR, 1.43; 95% CI, 1.20-1.70), and White (9.7% to 11.7%; aOR, 1.24; 95% CI, 1.13-1.35) participants. No changes were found in the prevalence of any cannabis outcome after RCL/after MCL among Black participants (past-year cannabis use: aOR, 1.07; 95% CI, 0.90-1.28). Excluding increasing odds of CUD among other (aOR, 1.45; 95% CI, 1.07-1.95), past-month daily cannabis use and CUD among cannabis users showed no increases after RCL/after MCL among any racial or ethnic groups. Moderate e-values suggest these findings could be partially explained by unmeasured confounding.

**Table 2.** Comparisons of Cannabis-Related Outcomes After vs Before Enactment of RCLs by Race and Ethnicity

Race or ethnicity	Past-year cannabis use			Past-month cannabis use		
	After MCL, %		After vs before RCL, aOR (95%CI)	After MCL, %		After vs before RCL, aOR (95% CI)
	Before RCL	After RCL		Before RCL	After RCL	
Non-Hispanic Black	14.8	15.8	1.07 (0.90-1.28)	9.3	10.5	1.13 (0.93-1.39)
Hispanic	11.7	15.0	1.33 (1.15-1.52)^a^	6.4	8.9	1.43 (1.22-1.69)^b^
Other^c^	14.8	18.5	1.31 (1.12-1.52)^d^	8.8	12.1	1.43 (1.20-1.70)^e^
Non-Hispanic White	16.6	19.4	1.21 (1.12-1.31)^f^	9.7	11.7	1.24 (1.13-1.35)^g^
**Daily cannabis use**						
Non-Hispanic Black	36.1	39.3	1.15 (0.79-1.66)	32.0	32.0	1.00 (0.70-1.44)
Hispanic	31.7	34.8	1.16 (0.85-1.56)	32.2	35.4	1.16 (0.88-1.52)
Other^c^	34.4	36.2	1.08 (0.79-1.49)	35.7	44.6	1.45 (1.07-1.95)^h^
Non-Hispanic White	37.6	38.0	1.02 (0.87-1.19)	32.5	30.7	0.92 (0.79-1.07)

Abbreviations: aOR, adjusted odds ratio; *DSM-5*, *Diagnostic and Statistical Manual of Mental Disorders* (Fifth Edition); MCL, medical cannabis law enactment; RCL, recreational cannabis law enactment.

^a^ Past-year cannabis use e-value: aOR, 1.57; lower limit 95% CI, 1.35.

^b^ Past-year cannabis use e-value: aOR, 1.68; lower limit 95% CI, 1.44.

^c^ Because small numbers of participants endorsed several of the listed categories, we recategorized Native American, Pacific Islander, Asian American, and more than 1 race as other race or ethnicity.

^d^ Past-year cannabis use e-value: aOR, 1.55; lower limit 95% CI, 1.31.

^e^ Past-month cannabis use e-value: aOR, 1.68; lower limit 95% CI, 1.42.

^f^ Past-year cannabis use e-value: aOR, 1.43; lower limit 95% CI, 1.31.

^g^ Past-month cannabis use e-value: aOR, 1.47; lower limit 95% CI, 1.32.

^h^ Past-year *DSM-5* cannabis use disorder among people that used cannabis in the past-year e-value: aOR, 1.69; lower limit 95% CI, 1.22.

### Racial and Ethnic Differences After RCL Enactment by Age Group

No changes were observed in any of the cannabis outcomes among participants aged 12 to 20 years of any racial or ethnic group and Black individuals of any age group (Table 3). Living in a state with enacted RCL was not associated with increased odds of daily cannabis use or *DSM-5* CUD among people who used cannabis of any racial/ethnic groups or age. Findings for CUD among cannabis users were similar when using *DSM-IV* criteria.

**Table 3.** Comparisons of Cannabis-Related Outcomes After vs Before Enactment of RCLs by Age Group and Race and Ethnicity

Age, y	Race	Past-year cannabis use			Past-month cannabis use		
		After MCL, %		After vs before RCL, aOR (95%CI)	After MCL, %		After vs before RCL, aOR (95% CI)
		Before RCL	After RCL		Before RCL	After RCL	
**All cannabis use**							
12-20	Non-Hispanic Black	22.0	19.7	0.87 (0.66-1.14)	12.2	11.8	0.97 (0.69-1.35)
	Hispanic	21.1	21.6	1.03 (0.85-1.25)	10.6	11.5	1.10 (0.85-1.39)
	Other^a^	22.0	22.9	1.06 (0.83-1.35)	12.2	13.2	1.09 (0.81-1.47)
	Non-Hispanic White	25.0	23.3	0.94 (0.83-1.07)	13.7	13.1	0.95 (0.82-1.11)
21-30	Non-Hispanic Black	19.3	23.9	1.31 (0.96-1.80)	12.4	14.8	1.23 (0.87-1.73)
	Hispanic	14.9	23.2	1.73 (1.38-2.16)^b^	8.2	13.6	1.76 (1.37-2.27)^c^
	Other^a^	20.1	24.8	1.32 (1.02-1.70)^d^	11.5	15.8	1.44 (1.08-1.92)^e^
	Non-Hispanic White	21.5	26.0	1.28 (1.13-1.45)^f^	12.5	15.4	1.28 (1.11-1.47)^g^
31-40	Non- Hispanic Black	14.5	13.6	0.93 (0.57-1.52)	9.9	10.8	1.10 (0.64-1.88)
	Hispanic	6.6	9.7	1.53 (0.99-2.36)	3.6	6.1	1.77 (1.04-3.01)^h^
	Other^a^	12.6	19.2	1.64 (1.11-2.44)^i^	7.8	13.9	1.90 (1.21-2.99)^j^
	Non-Hispanic White	13.6	16.7	1.27 (1.07-1.51)^k^	8.3	10.4	1.29 (1.06-1.58)^l^
≥41	Non-Hispanic Black	7.0	7.6	1.09 (0.74-1.60)	5.0	5.2	1.06 (0.68-1.66)
	Hispanic	4.0	7.4	1.93 (1.22-3.06)^m^	2.3	4.9	2.14 (1.24-3.72)^n^
	Other^a^	7.4	11.5	1.63 (1.09-2.45)^o^	5.1	8.4	1.70 (1.07-2.71)^p^
	Non-Hispanic White	6.4	9.1	1.46 (1.26-1.70)^q^	4.2	6.2	1.50 (1.26-1.79)^r^
**Daily cannabis use**							
12-20	Non-Hispanic Black	28.3	28.5	1.01 (0.51-2.02)	33.7	30.5	0.87 (0.48-1.56)
	Hispanic	29.7	34.0	1.22 (0.77-1.92)	41.6	45.2	1.16 (0.79-1.70)
	Other^a^	32.8	31.7	0.95 (0.54-1.67)	44.9	54.5	1.47 (0.90-2.41)
	Non-Hispanic White	34.2	31.1	0.87 (0.65-1.16)	41.7	40.0	0.93 (0.73-1.20)
21-30	Non-Hispanic Black	40.4	45.6	1.24 (0.70-2.21)	36.0	36.9	1.04 (0.60;1.78)
	Hispanic	33.1	37.1	1.19 (0.76-1.85)	30.7	38.0	1.46 (0.62-3.45)
	Other^a^	34.1	42.3	1.42 (0.87-2.32)	35.3	45.1	1.36 (0.47-3.94)
	Non-Hispanic White	41.3	41.7	1.02 (0.81-1.29)	32.1	30.2	0.92 (0.73-1.15)
31-40	Non-Hispanic Black	46.0	49.6	1.15 (0.43-3.08)	26.2	28.0	1.05 (0.38-2.96)
	Hispanic	32.1	28.3	0.83 (0.29-2.38)	19.4	15.3	0.75 (0.21-2.64)
	Other^a^	38.4	36.4	0.92 (0.41-2.08)	22.4	19.5	0.84 (0.33-2.15)
	Non-Hispanic White	37.7	42.5	1.22 (0.85-1.76)	19.1	16.2	0.82 (0.51-1.31)
≥41	Non- Hispanic Black	33.5	34.6	1.05 (0.44-2.50)	17.1	15.0	0.86 (0.30-2.53)
	Hispanic	32.1	33.9	1.08 (0.37-3.18)	17.7	5.4	0.27 (0.03-2.28)
	Other^a^	35.5	28.0	0.71 (0.29-1.75)	15.8	32.3	2.54 (1.00-6.44)
	Non-Hispanic White	34.8	36.5	1.08 (0.77-1.51)	12.8	14.6	1.06 (0.67-1.68)

Abbreviations: aOR, adjusted odds ratio; MCL, medical cannabis law enactment; RCL, recreational cannabis law enactment.

^a^ Because small numbers of participants endorsed several of the listed categories, we recategorized Native American, Pacific Islander, Asian American, and more than 1 race as other race or ethnicity.

^b^ Past-year cannabis use e-value: aOR, 1.96; lower limit 95% CI, 1.63.

^c^ Past-month cannabis use e-value: aOR, 1.98; lower limit 95% CI, 1.62.

^d^ Past-year cannabis use e-value: aOR, 1.56; lower limit 95% CI, 1.11.

^e^ Past-month cannabis use e-value: aOR, 1.69; lower limit 95% CI, 1.24.

^f^ Past-year cannabis use e-value: aOR, 1.52; lower limit 95% CI, 1.32.

^g^ Past-month cannabis use e-value: aOR, 1.52; lower limit 95% CI, 1.29.

^h^ Past-month cannabis use e-value: aOR, 1.99; lower limit 95% CI, 1.16.

^i^ Past-year cannabis use e-value: aOR, 1.88; lower limit 95% CI, 1.29.

^j^ Past-month cannabis use e-value: aOR, 2.10; lower limit 95% CI, 1.43.

^k^ Past-year cannabis use e-value: aOR, 1.51; lower limit 95% CI, 1.22.

^l^ Past-month cannabis use e-value: aOR, 1.53; lower limit 95% CI, 1.20.

^m^ Past-year cannabis use e-value: aOR, 2.12; lower limit 95% CI, 1.44.

^n^ Past-month cannabis use e-value: aOR, 2.29; lower limit 95% CI, 1.47.

^o^ Past-year cannabis use e-value: aOR, 1.87; lower limit 95% CI, 1.26.

^p^ Past-month cannabis use e-value: aOR, 1.93; lower limit 95% CI, 1.22.

^q^ Past-year cannabis use e-value: aOR, 1.71; lower limit 95% CI, 1.49.

^r^ Past-month cannabis use e-value: aOR, 1.75; lower limit 95% CI, 1.49.

Living in a state after RCL/after MCL was associated with increased odds of past-year cannabis use among other and White individuals aged 21 to 30 years (other: aOR, 1.32; 95% CI, 1.02-1.70; White: aOR, 1.28; 95% CI, 1.13-1.45), 31 to 40 years (other: aOR, 1.64; 95% CI, 1.11-2.44; White: aOR, 1.27; 95% CI, 1.07-1.51), and 41 years and older (other: aOR, 1.63; 95% CI, 1.09-2.45; White: aOR, 1.46; 95% CI, 1.26-1.70), the latter showing the highest aOR. Hispanic individuals had increased odds of past-year cannabis use after RCL/after MCL among those aged 21 to 30 years (aOR, 1.73; 95% CI, 1.38-2.16) and 41 years and older (aOR 1.93; 95% CI, 1.22-3.06), the latter showing the highest OR. Other, White, and Hispanic individuals aged 21 and older all had increased odds of past-month cannabis use after RCL/after MCL. While for most associations the e-values were large, the small e-values for the lower limit 95% CI suggests that unmeasured confounding may account for the observed association between after RCL/after MCL and past-year cannabis use among other individuals aged 21 to 30 and between after RCL/after MCL and past-month cannabis use among Hispanics aged 31 to 40 years.

## Discussion

This study evaluated the role of RCLs on cannabis-related outcomes by race and ethnicity overall and by age groups, focusing on the enactment of recreational laws beyond existing medical laws. Our findings indicate that the enactment of RCLs was followed by increases in the odds of past-year and past-month cannabis use among individuals aged 21 and older self-identifying as Hispanic, other, and non-Hispanic White, but not among non-Hispanic Black individuals or among individuals aged 12 to 20 years of any race or ethnicity. No changes were observed in the odds of past-month daily cannabis use among people who used cannabis for any race or ethnicity and by age group. We observed post-RCL increases in the odds of CUD among people that used cannabis among participants categorized as other, but this association was no longer observed after stratifying by age.

To our knowledge, this is the first study to explore racial and ethnic–specific and age-stratified associations between enacted RCLs and cannabis outcomes beyond MCLs. Disaggregating analyses by race and ethnicity is important as studies conducted prior to RCL enactment identified differential trends in cannabis outcomes over time by race and ethnicity. Furthermore, as one of the stated goals of cannabis legalization is to combat racial inequalities in cannabis legislation enforcement, it is critical to examine patterns of cannabis use in the context of persistent racial and ethnic disparities in cannabis arrests and incarceration.^9,10^ Our findings show that past-year and past-month cannabis use among non-Hispanic White respondents ages 21 years or older consistently increased following recreational cannabis legalization, while past-year and past-month use remained unchanged among non-Hispanic Black participants. No changes in use were observed for any racial or ethnic group among individuals aged 12 to 20, for whom cannabis use remains illegal. Nevertheless, Black people were still 1.72 times more likely to be arrested for cannabis possession in states that legalized cannabis before 2018,^9^ indicating that racist and discriminatory targeting of Black people persists despite changing policies.^9,10^ Given that a stated goal of RCL proponents^26^ is to combat racial inequities in cannabis prohibition enforcement, the connection between patterns of cannabis use and arrests by race and ethnicity warrants further attention.

While prior reports on national trends indicate greater increases in the odds of cannabis use among non-Hispanic Black populations than non-Hispanic White,^27,28^ these findings were mainly based upon data of use in most states with RCLs prior to enactment^27,28^; our results suggest that those increases are not attributable to recreational policy laws. Furthermore, we found no increases in the odds of past-month daily cannabis use among people that used cannabis in the past month, and only found increases in the odds of past-year *DSM-5* CUD^29^ among individuals in the other racial and ethnic subgroup with increased past-year cannabis use, a largely heterogeneous group comprising people from very different racial/ethnic backgrounds (ie, Asian Americans, Native American and Pacific Islanders, and those that reported more than 1 race). These results were not consistent when we stratified by age group and should be interpreted with caution and examined more closely in future studies. This is partially consistent with recent reports on trends in *DSM-5* CUD in the general population, although that work did not examine the role of RCL enactment.^17^ Longer-term studies are needed across all racial and ethnic groups to monitor whether or not the prevalence of daily cannabis use and CUD remain unchanged in the future. In particular, it may be too early to see increases in the odds of *DSM-5* CUD among people who use cannabis, since transition to CUD can occur only several years after regular cannabis use.^30,31^ Because *DSM-5* excluded “legal problems” as a disorder criterion, findings would not be attributable to potentially racialized changes in cannabis legalization enforcement.^29^

### Limitations

This study had several limitations that should be noted. First, because of small subpopulation sample sizes, we created an aggregate other race and ethnicity category, limiting the applicability of our findings to subpopulations included in this category. Second, because this study relied on self-reported cannabis use, social desirability about reporting use could potentially have differentially changed by race or ethnicity after RCL. In NSDUH, the use of computer-assisted self-interviewing reduces these concerns.^32^ Third, the NSDUH excluded unhomed individuals who do not live in shelters or who reside in institutions and correctional settings, which could underestimate the prevalence of cannabis outcomes. Fourth, we did not explore variations in policy provisions (eg, number of legal dispensaries, cultivation, and consumption restrictions). This study also has several strengths including its large, nationally representative samples across multiple years, data self-reported by racial and ethnic groups, and a survey design that provided for accurate state-level estimates.

## Conclusions

This study contributes to our understanding of racial and ethnic changes in cannabis use that occur after the legalization of adult marijuana use in the US beyond medical cannabis laws.^33,34,35^ Changes in legalization may contribute to an increase in use specifically among non-Hispanic White, Hispanic, and other adults. To ensure that the enactment of recreational cannabis laws truly contributes to greater racial and ethnic equity and adheres to antiracist policies, monitoring both unintended and intended consequences attributable to recreational cannabis laws and their associated policy provisions by race and ethnicity is needed.

## Notes

Supplement. **eAppendix.** Example Code on the Time-Varying Indicator of Cannabis Legalization Status **eTable 1.** Dates when RCLs Became Effective **eTable 2.** Mapping of *DSM-5* and *DSM-IV* Cannabis Use Disorder Criteria
