# Error in Tables

**DOI:** 10.1001/jamanetworkopen.2021.36454

**Published:** 2021-10-27

In the Original Investigation titled “Racial and Ethnic Differences in Cannabis Use Following Legalization in US States With Medical Cannabis Laws,”^1^ published September 27, 2021, there were errors in the column headings in Tables 2 and 3. Subheadings have been added to clarify these results. This article has been corrected.^1^
