# Supplementary material for: Racial and Ethnic Differences in Cannabis Use Following Legalization in US States With Medical Cannabis Laws
Source: JAMA Netw Open. 2021 Sep 27;4(9):e2127002. doi: 10.1001/jamanetworkopen.2021.27002 (PMC8477268; doi:10.1001/jamanetworkopen.2021.27002)
Supplement: Supplement. — eAppendix. Example Code on the Time-Varying Indicator of Cannabis Legalization Status eTable 1. Dates when RCLs Became Effective eTable 2. Mapping of DSM-5 and DSM-IV Cannabis Use Disorder Criteria [file jamanetwopen-e2127002-s001.pdf]

Supplemental Online Content

Martins SS, Segura LE, Levy NS, et al. Racial and ethnic differences in cannabis use following legalization in US states with medical cannabis laws. *JAMA Netw Open.* 2021;4(9):e2127002. doi:10.1001/jamanetworkopen.2021.27002

eAppendix. Example Code on the Time-Varying Indicator of Cannabis Legalization Status

eTable 1. Dates when RCLs Became Effective

eTable 2. Mapping of *DSM-5* and *DSM-IV* Cannabis Use Disorder Criteria

This supplemental material has been provided by the authors to give readers additional information about their work.

© 2021 Martins SS et al. *JAMA Network Open.*

eAppendix. Example Code on the Time-Varying Indicator of Cannabis Legalization Status

Step 0. Loading libraries, files, and datasets. Merging files.

Load libraries used in this session

```
library(tidyverse)
```

Load file containing effective RCL and MCL dates compiled from existing literature, Marijuana Policy Project, ProCon, The National Alliance for Model State Drug Laws, The National Conference of State Legislatures, The Prescription Drug Abuse Policy System, and the Alcohol Policy Information System.

```
policyDates <- read.csv("CannabisPolicyDates.csv")
```

Load NSDUH dataset

```
nsduh <- load("NSDUH.RData")
```

Join policyDates file to NSDUH dataset by state FIPS code

```
dataset <- left_join(nsduh, policyDates, by = c("states_FIPS"))
```

Step 1. Create a time-fixed indicator of MCL/RCL status used for descriptive analysis in TABLE 1.

This variable indicates whether an individual lived in a state that never had an effective RCL or MCL law (never_mcl_rcl), or ever had an effective MCL (ever_mcl), or ever having an effective RCL (ever_rcl).

```
dataset <- dataset %>%

  mutate(never_mcl_rcl = factor(ifelse(is.na(mcl_effective_date) == T &

                                  is.na(rcl_effective_date) == T, 1, 0),

                                labels = c("Ever MCL & RCL", "Never MCL & RCL")),

         ever_mcl = factor(ifelse(is.na(mcl_effective_date) == F, 1, 0),

                          labels = c("Never MCL", "Ever MCL")),

         ever_rcl = factor(ifelse(is.na(rcl_effective_date) == F, 1, 0),

                          labels = c("Never RCL", "Ever RCL")))
```

Step 2. Create a time-varying indicator of living in a state that had an effective MCL or RCL at the time of interview.

For each survey year, we compared individuals' interview date (refdate) to MCL/RCL effective date to determine RCL/MCL time-varying status. For example, if in year X interview date was < than RCL effective date then the individual lived in a state before RCL was effective in year X. Conversely if refdate > than RCL effective date then the individual lived in a state after RCL was

© 2021 Martins SS et al. *JAMA Network Open.*

effective in year X. This variable was used for our main analysis (Tables 2 and 3). The contrast of interest was After RCL, After MCL vs Before RCL, After MCL.

```
dataset <- dataset %>%

  mutate(mcl_status = factor(ifelse(is.na(mcl_effective_date) == T, 1,

                           ifelse(is.na(mcl_effective_date) == F &

                                  refdate <= mcl_effective_date, 2,

                                 ifelse(is.na(mcl_effective_date) == F &

                                        refdate > mcl_effective_date, 3, 9999))),

                           labels = c("Never MCL", "Before MCL", "After MCL")),

         rcl_status = factor(ifelse(is.na(rcl_effective_date) == T, 1,

                           ifelse(is.na(rcl_effective_date) == F &

                                  refdate <= rcl_effective_date, 2,

                                 ifelse(is.na(rcl_effective_date) == F &

                                        refdate > rcl_effective_date, 3, 9999))),

                           labels = c("Never RCL", "Before RCL", "After RCL")),

         mcl_rcl_status = factor(ifelse(is.na(mcl_effective_date) == T &

                                     is.na(rcl_effective_date) == T, 1,

                                   ifelse(is.na(mcl_effective_date) == F &

                                          is.na(rcl_effective_date) == T &

                                          refdate <= mcl_effective_date, 2,

                                   ifelse(is.na(mcl_effective_date) == F &

                                          is.na(rcl_effective_date) == T &

                                          refdate > mcl_effective_date, 3,

                                   ifelse(is.na(mcl_effective_date) == F &

                                          is.na(rcl_effective_date) == F &

                                          refdate <= mcl_effective_date &

                                          refdate <= rcl_effective_date, 4,

                                   ifelse(is.na(mcl_effective_date) == F &

                                          is.na(rcl_effective_date) == F &

                                          refdate > mcl_effective_date &

                                          refdate <= rcl_effective_date, 5,

                                   ifelse(is.na(mcl_effective_date) == F &
```

© 2021 Martins SS et al. *JAMA Network Open.*

```
                            is.na(rcl_effective_date) == F &
                            refdate > mcl_effective_date &
                            refdate > rcl_effective_date, 6, 9999)))))),
                  labels = c("Never MCL & RCL",
                             "Before MCL, Never RCL",
                             "After MCL, Never RCL",
                             "Before MCL, Before RCL",
                             "After MCL, Before RCL",
                             "After MCL, After RCL")))
```

© 2021 Martins SS et al. *JAMA Network Open.*

eTable 1. Dates When RCLs Became Effective

State	RCL Effective Date
Alaska	2/24/15
Arizona	NA
Arkansas	NA
California	11/9/16
Colorado	12/10/12
Connecticut	NA
Delaware	NA
Florida	NA
Hawaii	NA
Illinois	NA
Louisiana	NA
Maine	1/30/17
Maryland	NA
Massachusetts	12/15/16
Michigan	12/6/18
Minnesota	NA
Missouri	NA
Montana	NA
Nevada	1/1/17
New Hampshire	NA
New Jersey	NA
New Mexico	NA
New York	NA
North Dakota	NA
Ohio	NA
Oklahoma	NA
Oregon	3/29/16
Pennsylvania	NA

© 2021 Martins SS et al. *JAMA Network Open.*

Rhode Island	NA
Utah	NA
Vermont	7/1/18
Washington	12/6/12
District of Columbia	2/26/15
West Virginia	NA

Sources: 33 Legal Medical Marijuana States and DC: Laws, Fees, and Possession Limits;[28] Legal Recreational Marijuana States and DC: Cannabis Laws with Possession and Cultivation Limits;[1] State by State MML Report;[27] The National Alliance for Model State Drug Laws;[29] The National Conference of State Legislatures;[30] The Prescription Drug Abuse Policy System;[31] and the Alcohol Policy Information System.[32]

© 2021 Martins SS et al. *JAMA Network Open.*

eTable 2. Mapping of *DSM-5* and *DSM-IV* Cannabis Use Disorder Criteria

**DSM-5 Cannabis Use Disorder Criteria**	**DSM-IV Cannabis Use Disorder Criteria**
Criterion 1: Using cannabis is larger amounts or over a longer period than was intended	*Dependence Criterion 3*: During the past 12 months, did you try to set limits on how often or how much marijuana or hashish you would use? Were you able to keep to the limits you set, or did you often use marijuana or hashish more than you intended to?
Criterion 2: Persistent desire or unsuccessful efforts to cut down or control cannabis use	*Dependence Criterion 4*: During the past 12 months, did you want to or try to cut down or stop using marijuana or hashish? During the past 12 months, were you able to cut down or stop using marijuana or hashish every time you wanted to or tried to?
Criterion 3: A great deal of time is spent in activities necessary to obtain cannabis, use cannabis, or recover from its effects	*Dependence Criterion 5*: During the past 12 months, was there a month or more when you spent a lot of your time getting or using marijuana or hashish? During the past 12 months, was there a month or more when you spent a lot of your time getting over the effects of the marijuana or hashish you used?
Criterion 4: Craving, or a strong desire or urge to use cannabis	*Not assessed*
Criterion 5: Recurrent cannabis use resulting in a failure to fulfill major role obligations at work, school, or home	*Abuse Criterion 1*: Sometimes people who use marijuana or hashish have serious problems at home, work or school — such as: neglecting their children, missing work or school, doing a poor job at work or school, losing a job or dropping out of school. During the past 12 months, did using marijuana or hashish cause you to have serious problems like this either at home, work, or school?
Criterion 6: Continued cannabis use despite having persistent or recurrent social or interpersonal problems caused or exacerbated by the effects of cannabis	*Abuse Criterion 4*: During the past 12 months, did you have any problems with family or friends that were probably caused by your use of marijuana or hashish? Did you continue to use marijuana or hashish even though you thought it caused problems with family or friends?
Criterion 7: Important social, occupational, or recreational activities are given up or reduced because of cannabis use	*Dependence Criterion 6*: This question is about important activities such as working, going to school, taking care of children, doing fun things such as hobbies and sports, and spending time with friends and family. During the past 12 months, did using marijuana or hashish cause you to give up or spend less time doing these types of important activities?
Criterion 8: Recurrent cannabis use in situations in which it is physically hazardous	*Abuse Criterion 2*: During the past 12 months, did you regularly use marijuana or hashish and then do

	something where using marijuana or hashish might have put you in physical danger?
Criterion 9: Cannabis use is continued despite knowledge of having a persistent or recurrent physical or psychological problem that is likely to have been caused or exacerbated by cannabis	*Dependence Criterion 7*: During the past 12 months, did you have any problems with your emotions, nerves, or mental health that were probably caused or made worse by your use of marijuana or hashish? Did you continue to use marijuana or hashish even though you thought it was causing you to have problems with your emotions, nerves, or mental health?<br>During the past 12 months, did you have any physical health problems that were probably caused or made worse by your use of marijuana or hashish? Did you continue to use marijuana or hashish even though you thought it was causing you to have physical problems?
Criterion 10: Tolerance	*Dependence Criterion 1*: During the past 12 months, did you need to use more marijuana or hashish than you used to in order to get the effect you wanted?<br>OR<br>During the past 12 months, did you notice that using the same amount of marijuana or hashish had less effect on you than it used to?
Criterion 11: Withdrawal	*Not assessed*

© 2021 Martins SS et al. *JAMA Network Open.*
